# Solid-state fermented brewer's spent grain enzymatic extract increases in vitro and in vivo feed digestibility in European seabass

**DOI:** 10.1038/s41598-021-02393-x

**Published:** 2021-11-25

**Authors:** Helena Fernandes, Francisco Moyano, Carolina Castro, José Salgado, Francisca Martínez, María Aznar, Nelson Fernandes, Patrícia Ferreira, Margarida Gonçalves, Isabel Belo, Aires Oliva-Teles, Helena Peres

**Affiliations:** 1 Department of Biology, Faculty of Sciences of University of Oporto (FCUP), Porto, Portugal; 2 Interdisciplinary Centre of Marine and Environmental Research (CIIMAR), Matosinhos, Portugal; 3 Department of Biology and Geology, University of Almería, Almería, Spain; 4 Centre of Biological Engineering (CEB), University of Minho, Campus de Gualtar, 4710-057 Braga, Portugal

**Keywords:** Animal physiology, Ichthyology

## Abstract

Brewer’s spent grain (BSG) is the largest by-product originated from the brewery industry with a high potential for producing carbohydrases by solid-state fermentation. This work aimed to test the efficacy of a carbohydrases-rich extract produced from solid-state fermentation of BSG, to enhance the digestibility of a plant-based diet for European seabass (*Dicentrarchus labrax*). First, BSG was fermented with *A. ibericus* to obtain an aqueous lyophilized extract (SSF-BSG extract) and incorporated in a plant-based diet at increasing levels (0—control; 0.1%, 0.2%, and 0.4%). Another diet incorporating a commercial carbohydrases-complex (0.04%; Natugrain; BASF) was formulated. Then, all diets were tested in in vitro and in vivo digestibility assays. In vitro assays, simulating stomach and intestine digestion in European seabass, assessed dietary phosphorus, phytate phosphorus, carbohydrates, and protein hydrolysis, as well as interactive effects between fish enzymes and dietary SSF-BSG extract. After, an in vivo assay was carried out with European seabass juveniles fed selected diets (0—control; 0.1%, and 0.4%). In vitro digestibility assays showed that pentoses release increased 45% with 0.4% SSF-BSG extract and 25% with Natugrain supplemented diets, while amino acids release was not affected. A negative interaction between endogenous fish enzymes and SSF-BSG extract was observed in both diets. The in vivo digestibility assay corroborated in vitro data. Accordingly, the dietary supplementation with 0.4% SSF-BSG increased the digestibility of dry matter, starch, cellulose, glucans, and energy and did not affect protein digestibility. The present work showed the high potential of BSG to produce an added-value functional supplement with high carbohydrases activity and its potential contribution to the circular economy by improving the nutritional value of low-cost and sustainable ingredients that can be included in aquafeeds.

## Introduction

Fish meal is one of the most expensive ingredients of aquafeeds and is still used as the main protein source for some particular fish species, namely for carnivorous fish. Several more sustainable and economical alternative protein sources to fish meal have been used, including plant ingredients^1^. However, the presence of antinutritional factors, low digestibility, and insufficient levels of some essential nutrients (namely amino acids and fatty acids) has limited the incorporation of plant ingredients in aquafeeds^1^. The presence of significant levels of indigestible carbohydrates, mainly non-starch polysaccharides (NSPs), is one of the main restrictions on their use in aquafeeds.

NSPs are structural constituents of the cell walls of plant feedstuffs, and their presence and composition vary according to the plant source^2^. Depending on their composition, dietary NSPs differently impact fish physiology, generally impairing feed utilization and growth performance^3^. It has been reported that supplementation of plant-based diets with exogenous carbohydrases, namely non-starch carbohydrases, increases diet digestibility, reduces nutrient excretion, and increases feed efficiency^4–6^. Carbohydrases are a wide group of enzymes, including cellulases, xylanases, and hemicellulases, which can hydrolyze NSPs into lower molecular weight polysaccharides and oligosaccharides^7^.

More than 80% of the global economic market of carbohydrases is dominated by xylanase and glucanase, but other enzymes are also available^4,8^. Special attention has been given to cellulose hydrolyzing enzymes, i.e., exoglucanases and endoglucanases, releasing cellobiose and glucose, respectively^9^. The potential of these enzymes to enhance the digestibility of carbohydrates in plant-based diets is well documented in terrestrial animals^10,11^ but it still needs further research in aquaculture nutrition^4^.

Solid-state fermentation (SSF) is a white biotechnological process that may be used to obtain carbohydrases through the hydrolysis of adequate solid substrates by selected microorganisms. SSF differs from submerged fermentation (SmF) as it occurs with little or no free water, is suitable for filamentous fungi growth, and is a cost-effective and eco-friendly process^12^. SmF is a liquid-state fermentation, commonly uses bacteria and allows easy final product recovery through filtration or centrifugation^13^. Even though SmF is the preferred process for producing enzymes at an industrial scale^14^, SSF presents some advantages over SmF, including lower susceptibility to contamination, microorganisms less vulnerable to inactivation by the substrate, and higher enzymatic productivity with higher specificity and activity^15^.

The production of enzymes with commercial interest through SSF, such as proteases, cellulases, xylanases, lipases, and phytases, can be modulated mainly by the microorganism species and type of substrate used in the fermentative process^16^. Carbohydrases are usually produced from lignocellulosic-rich by-products of agricultural origin^17^, as brewer’s spent grain (BSG). BSG is the main by-product resulting from the brewery industry, averaging 20 kg of BSG per 100 L of beer^18^. BSG is produced during the malting stage of beer manufacturing and is composed of barley grain husks, smaller parts of the pericarp, and endosperm. BSG possesses high hemicellulose and cellulose content (30–50% w/w), relatively high protein levels (19–30% w/w), residual levels of starch and hops, and a variable quantity of phenolic compounds^18,19^. Even though these nutritional characteristics make BSG a low-value feedstuff only suitable for ruminants nutrition^18^, BSG is an excellent substrate for SSF. Indeed, during SSF, the lignocellulosic structure and the free sugars available of BSG are used as a nutrient source and solid porous support matrix for fungal growth^20^. BSG has already been used to produce enzymes such as laccases^21^, xylanases^22–24^, cellulases^24,25^, and multi-complex cocktails of enzymes^26^. However, the potential application of a carbohydrases-enriched extract produced by SSF of BSG in aquafeeds has not yet been studied.

Although some research has been made to assess how plant-based diets supplementation with carbohydrases affects fish growth and feed utilization, to the best of our knowledge, there are no studies regarding the efficacy of enzymes produced through SSF of BSG in aquafeeds. The present work aimed to produce a carbohydrases-enriched extract by SSF of BSG and to evaluate its potential as an enzymatic feed additive using European seabass (*Dicentrarchus labrax*) as model species.

Firstly, BSG was fermented with *A. ibericus* to obtain an aqueous lyophilized extract (SSF-BSG extract) and incorporated in a plant-based diet at increasing levels. Then, the in vitro digestibility assays were performed to assess, the efficacy of SSF-BSG extract to improve the bioavailability of dietary phosphorus (P), phytate P, carbohydrates, and protein, as well as the interactions between fish digestive enzymes (endoenzymes) and dietary SSF-BSG extract enzymes. In vitro techniques that mimic in vivo fish digestion are methods based on the 3R’s approach (animal reduced use), successfully used to predict nutritional responses and to explain interactions between the feed matrix, additives, and fish enzymes^27,28^. Finally, using the results of in vitro assays as a reference, some of the most interesting diets were selected and tested in an in vivo digestibility trial. Results of both types of assays were correlated and discussed.

## Materials and methods

This study was approved by the CIIMAR Animal Welfare Committee (ORBEA) and by the Portuguese National Authority for Animal Health (DGAV); reference ORBEACIIMAR_27_2019) and was carried out according to ARRIVE guidelines^29^. Experiments were directed by trained scientists (Functions A, B, C & D defined in article 23 of European Union Directive 2010/63) and conducted following the Federation of Laboratory Animal Science Association (FELASA) recommendations for experiments in laboratory animals^30^ and the EU Directive (2010/63/EU) on the protection of animals for scientific purposes.

### Solid-state fermentation of brewer’s spent grain

Brewer’s spent grain (BSG) was provided by UNICER S.A. (Porto, Portugal). BSG proximal composition is presented in Table 1. SSF of BSG was performed with *Aspergillus ibericus* (MUM 03.49), maintained at 4 °C in Potato Dextrose Agar medium until utilization. This fungus was selected as it has GRAS (Generally Regarded As Safe) status and a high ability to produce carbohydrases, namely xylanase and cellulase. The SSF was carried out using 4 trays (43 × 33 × 7 cm) as bioreactors. In each tray, 400 g of dried BSG (bed height 2.5 cm) was inoculated with a spore’s suspension of *A. ibericus* with 10^6^ spores’ concentration. BSG humidity was then adjusted to 75%, followed by incubation at 25 °C for 7 days. At the end of SSF, water-soluble compounds of fermented BSG were recovered. For that, the fermented BSG was mixed with distilled water (1:5 w/v) under constant agitation for 30 min^31^, sieved (0.45 µm pore-size filter), centrifuged (7000*g* for 5 min), and the supernatant (crude extract) was recovered and lyophilized for 48 h. The xylanase and cellulase activities of the lyophilized fermented BSG extract (SSF-BSG extract) are presented in Table 1.

**Table 1:** Brewer’s spent grain composition and enzymatic activities of SSF-BSG extract and experimental diets.

Composition (% dry matter)	Moisture	Reducing sugars (mg sugars g^−1^)	Protein	Cellulose	Hemicellulose	Lignin
BSG	4.3	6.7	25.8	21.2	23.8	15.0

Enzymatic activity (U kg^−1^ diet)	Cellulase	Xylanase	β-glucosidase	Phytase	Protease	
SSF-BSG extract	1343	15,885	–	–	–	
**Experimental diets**						
Control	270	309	330	280	92	
BSG0.1	1110	1590	330	652	646	
BSG0.2	1910	5538	510	717	1824	
BSG0.4	5075	10,153	830	1050	1686	
BSG0.4 +	4420	9955	820	989	1740	
NAT	1220	4590	710	1036	–	

BSG: Brewer’s spent grain.

SSF-BSG extract: lyophilized extract obtained after solid-state fermentation of BSG with *Aspergillus ibericus.*

### Diets formulation

Six diets were formulated to be isoproteic (48% crude protein) and isolipidic (16% crude lipids), containing 15% of fishery products as protein source (fish meal and fish protein concentrate) and fish oil as the main lipid source. Chromium oxide was used as a digestibility marker. SSF-BSG extract was included in the diets at 0, 0.1, 0.2, and 0.4% of dry matter (control, BSG0.1, BSG0.2, and BSG0.4 diets, respectively), corresponding to a cellulase supplementation of 0, 1000, 2000, and 4000 U g^−1^ of diet (DM basis). Another BSG0.4 diet was formulated, including a commercial catalyzer (Silica+; CERESCO NUTRITION; inclusion level 0.02% of the diet) and a plant-derived detoxifier (Ena-Detox; EUROPEAN NATURAL ADDITIVES, inclusion level 0.07% of the diet) (diet BSG0.4+). For comparison purposes, a diet supplemented with a commercial carbohydrases-complex (*endo*-1,4-β-xylanase, and *endo*-1,4-β-glucanase complex; Natugrain TS, BASF, Germany) was formulated with a cellulase activity of 4000 U g^−1^ of diet (DM basis; NAT diet). The diets were produced as pellets according to Magalhães et al*.*^5^. The proximate composition of the control diet is presented in Table 2.

**Table 2:** Composition and proximate analysis of all experimental diets.

Diets	Control
**Ingredients (% DM)**	
Fish meal^a^	9.8
Soluble fish protein concentrate^b^	4.9
Wheat gluten meal^c^	18.6
Soybean meal^d^	9.8
Rice bran meal^e^	9.8
Sunflower meal^f^	8.8
Rapeseed meal^g^	7.8
Wheat meal^h^	5.4
Hemoglobin AP310^i^	4.9
Taurine^j^	0.5
l-Lysine^j^	0.6
dl-Methionine^j^	0.2
Fish oil	12.3
Vitamin premix^k^	1
Choline chloride (50%)	0.5
Mineral premix^l^	1
Binder^m^	1
Betain^n^	0.2
Shrimp hydrolysed^o^	1.2
CaHPO_4_	1
Chromium oxide	0.5
**SSF-BSG extract**^**p**^	0.1–0.4
Ena-detox^q^	0.07
Silicon dioxide^r^	0.02
Natugrain TS^s^	0.04
**Proximate composition (%)**	
Moisture	7.1
Protein	47.9
Lipids	16.3
Ash	6.8
Energy (kJ g^−1^)	24.1
Starch	13.9
Hemicellulose	5.8
Cellulose	4.3
Lignin	2.6

CP: Crude protein; CL: Crude lipids; DM: Dry matter.

^a^Steam Dried LT-FM, Pesquera Centinela, Chile (CP: 71.2%; CL 11.1%).

^b^Sopropèche G, France (CP: 79.7%; CL: 18.4%DM).

^c^Sorgal, S.A. Ovar, Portugal (CP: 86.9%; CL: 1.0%).

^d^Sorgal, S.A. Ovar, Portugal (CP: 53.8%; CL: 1.8%).

^e^Sorgal, S.A. Ovar, Portugal (CP: 14.1%; CL: 1.0%).

^f^Sorgal, S.A. Ovar, Portugal. (CP: 29.0%; CL: 1.8%).

^g^Sorgal, S.A. Ovar, Portugal. (CP: 41.5%; CL: 1.9%).

^h^Sorgal, S.A. Ovar, Portugal. (CP: 12.4%; CL: 0.9%).

^i^APC Europe S.A.S.A. (CP: 94.5%; CL: 0.4%).

^j^Feed-grade taurine, l-lysine and dl-Methionine, Sorgal, S.A. Ovar, Portugal.

^k^Vitamins (mg kg^−1^ diet): retinol, 18,000 (IU kg^−1^ diet); calciferol, 2000 (IU kg^−1^ diet); alpha tocopherol, 35; menadion sodium bis., 10; thiamin, 15; riboflavin, 25; Ca pantothenate, 50; nicotinic acid, 200; pyridoxine, 5; folic acid, 10; cyanocobalamin, 0.02; biotin, 1.5; ascorbyl monophosphate, 50; inositol, 400.

^l^Minerals (mg kg^−1^ diet): cobalt sulphate, 1.91; copper sulphate, 19.6; iron sulphate, 200; sodium fluoride, 2.21; potassium iodide, 0.78; magnesium oxide, 830; manganese oxide, 26; sodium selenite, 0.66; zinc oxide, 37.5; dicalcium phosphate, 8.02 (g kg^−1^ diet); potassium chloride, 1.15 (g kg^−1^ diet); sodium chloride, 0.4 (g kg^−1^ diet).

^m^LIPTOSA, Spain.

^n^Sorgal, S.A. Ovar, Portugal.

^o^Sorgal, S.A. Ovar, Portugal. (CP: 69.8%; CL: 12.1%).

^p^Lyophilized extract obtained after solid-state fermentation of BSG with *Aspergillus ibericus* (xylanase and cellulase activities are, respectively, 15885 and 1343 U g^−1^ lyophilized extract). Incorporation levels: BSG0.1 diet—0.1%; BSG0.2 diet—0.2%; BSG0.4 diet—0.4%; BSG0.4 + diet—0.4%.

^q^Plant extract additive EUROPEAN NATURAL ADDITIVES, Madrid, Spain. Included in BSG0.4 + diet.

^r^Silica+, CERESCO NUTRITION, Quebec, Canada. Included in BSG0.4+ diet.

^s^Natugrain TS, BASF, Germany (2500 and 5600 U g^−1^ of cellulase and xylanase activity, commercial information). Included in NAT diet.

**Figure 1:**
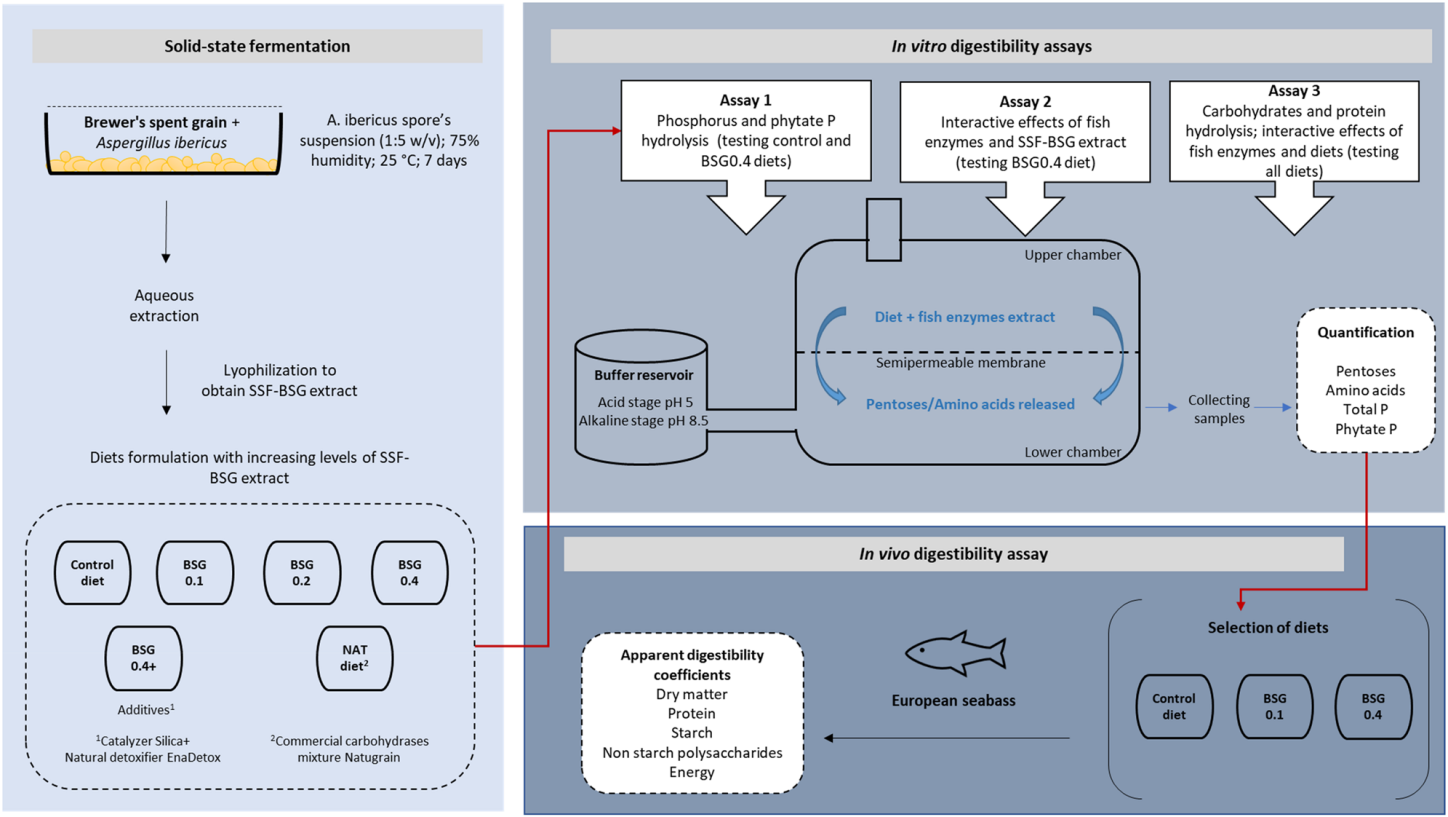
Flow chart of the experimental design.

In vitro and in vivo trials performed are schematically indicated in Fig. 1 and described below.

### In vitro digestibility assays

Three different in vitro assays were carried out to determine the:

- *Assay 1* > effect of SSF-BSG extract on phosphorus (P) availability (total and phytate P) of control and BSG0.4 diets, measured by total P and phytate P release.
- *Assay 2* > interactive effects of SSF-BSG extract and fish endoenzymes on the carbohydrates hydrolysis of BSG0.4 diet, measured by pentoses release. The conditions tested included: (i) active BSG0.4 diet and inactive fish enzymes; (ii) inactive BSG0.4 diet and active fish enzymes; and (iii) inactive BSG0.4 diet and inactive fish enzymes.
- *Assay 3* > effect of SSF-BSG extract, SSF-BSG extract plus catalyzer and detoxifier, or Natugrain on carbohydrates and protein hydrolysis of control, BSG0.1, BSG0.2, BSG0.4, BSG0.4+, and NAT diets, measured by pentoses and amino acids release with active or inactive fish enzymes, to estimate the interaction between fish enzymes and dietary SSF-BSG extract or NAT activity.

The fish enzyme extracts used in all the in vitro digestibility assays were obtained from full-fed European seabass juveniles as described by Morales and Moyano^27^. Briefly, fish were starved for 6 h prior to sampling and sacrificed by immersion in ice-cold water. Fish extracts were obtained after individual homogenization of the stomach and proximal intestine with pyloric caeca with distilled water (1:10 w/v), followed by centrifugation (12,000 g, 3 °C, 15 min). Fish extracts were lyophilized and resuspended when needed. The fish enzymes extract:diet ratio used was 13,000 U g^−1^ and 6800 U g^−1^ for acid and alkaline digestion. This ratio was defined considering the average total acid and basic proteases produced by European seabass (with circa 50 g body weight) fed a 45% crude protein diet at a ration level of 3% of body weight.

All in vitro digestibility assays were performed using bioreactors modified from those described by Morales and Moyano^27^. The mixture of fish enzyme extracts and the desired diet is placed in the upper chamber, and the released products of hydrolysis (amino acids, pentoses, P, and others) pass through the membrane into the lower chamber, being recovered at different time intervals during the reaction time. Acid and alkaline stages of the hydrolysis were carried out at pH 5 and 8.5, respectively, according to the average pH measured in the stomach and intestine of fed European seabass (50 g average weight)^32^.

All in vitro digestibility assays were run in triplicate for each diet. At the beginning of each run, the diet, fish acid enzymatic extract, and acid buffer (0.2 M citrate buffer with NaCl 50 mM, pH 5) were placed in the upper chamber and maintained under continuous agitation (270 rpm) for 2 h. During this time, three samples were taken from the upper and lower chambers at 30 min, 1 h, and 2 h. Samples taken from the upper chamber were centrifuged (14,000 *g*, 4 °C, 15 min), and the supernatant was stored at − 20 °C for determination of residual enzyme activities. After acid digestion, the pH of the upper chamber was adjusted to 8.5 with NaOH 6 N, and the crude alkaline enzymatic extract was added. Subsequently, an alkaline buffer (0.01 M Borax-Boric buffer with 20 mM CaCl_2_ and 45 µM taurocholate, pH 8.5) was added in the lower chamber to begin the alkaline digestion. Alkaline hydrolysis was maintained for 4 h, and a sample was taken every hour from the lower chamber. Moreover, at the end of the digestion, a sample was also collected from the upper chamber. The entire digestion process (6 h) was carried out at 25 °C, being stopped by adding trichloroacetic acid (20%) before the last sample collection.

Specific assays aimed to evaluate the effect of SSF-BSG extract or commercial product (NAT) in the absence of active fish enzymes were carried out after inactivating fish enzyme extracts by heating at 100 °C for 30 min.

### In vivo digestibility assay

Based on the in vitro assays results and to reduce the number of fish used in in vivo assays, the control, BSG0.1, and BSG0.4 diets were selected to be tested in an in vivo digestibility assay with European seabass juveniles. The assay was performed in a recirculating aquaculture system (RAS) equipped with 9 fiberglass tanks (60 l water capacity each) provided with a feces settling column connected to the outlet, designed according to Cho et al.^33^. During the assay, a constant water flow of 5 l min^−1^ was kept, the water temperature was regulated to 22 ± 1 °C, salinity averaged 32 ± 1 ‰, dissolved oxygen averaged 92% of saturation, ammonia and nitrites levels were kept below 0.02 mg ml^−1^, and photoperiod was adjusted to 12 L: 12 D.

Nine groups of 15 fish (initial body weight of 22 ± 1 g) were randomly distributed to each tank, and each experimental diet was randomly assigned to triplicate groups. Fish were fed by hand, until apparent satiety, twice a day, 7 days a week, for 64 days. During the last 30 days of feeding, feces accumulated in each settling column were collected daily before the first meal. Feces were then centrifuged at 3000 *g* for 15 min, pooled for each tank, and stored at − 20 °C until further analysis. After the last daily meal, the tanks, pipes, and settlings columns were cleaned to remove remaining feces and uneaten feed. Apparent digestibility coefficients (ADC) of dry matter, protein, gross energy, starch, cellulose, hemicellulose, glucans, xylan, arabinan, and lignin of the experimental diets were calculated as follows:

$$ADC \, \% = \left[ {1 - \left( {Dietary{\text{ Cr}}_{2} {\text{O}}_{3} \;level \, \times \, Feces \, nutrient \, or \, energy \, level/Feces{\text{ Cr}}_{2} {\text{O}}_{3} \;level \, \times \, Dietary \, nutrient \, or \, energy \, level} \right)} \right] \times 100$$

### Physical–chemical analysis

#### Enzymatic activity analysis

Acid protease activity on fish stomach extracts used in the in vitro assays was measured^34^ using 0.5% w/v hemoglobin in a glycine–HCl buffer (100 mM, pH 2.0) as substrate. Alkaline protease activity in intestinal extracts was measured^35^ using 1% w/v casein dissolved on Tris–HCl buffer (100 mM + 10 mM CaCl_2_, pH 8) as substrate. For both assays, one unit of activity was defined as 1 µg of tyrosine released per minute. Xylanase and cellulase activities were measured in SSF-BSG extract and experimental diets, while β-glucosidase, phytase, proteases, and lipase activities were measured only in the experimental diets. Xylanase (*endo*-1,4-β-xylanase), cellulase (*endo*-1,4-β-glucanase), and β-glucosidase were measured according to Fernandes et al.^36^.

Phytase activity was assessed^37^ using sodium phytate in acetate buffer (0.2 M, pH 4.5) as substrate, and the activity was defined as the enzyme needed to release 1 µM of inorganic phosphate.

Protease and lipase activities were measured according to Charney and Tomarelli^38^ and Gomes et al.^39^, respectively.

#### Analysis of products released during in vitro assays

In the in vitro digestibility assays, variations in phytate P, pentoses, and amino acids were measured as indicators of the hydrolysis of phytic acid, non-starch polysaccharides, and protein, respectively.

Phytate P was determined according to Haug and Lantzsch^40^. Pentoses were measured according to Douglas^41^. Total amino acids were quantified according to Church et al.^42^.

### Chemical analyses of ingredients, experimental diets, and feces

Chemical analyses were performed according to the Association of Official Analytical Chemists methods (AOAC79). Cellulose, hemicellulose, and lignin contents were determined according to Fernandes et al*.*^36^.

### Statistical analysis

Before analysis, normality and homogeneity of variances were verified using the Shapiro–Wilk test. When necessary, data were normalized using log-transformation or arc-sin square-root transformation for percentage values.

In vitro assays data of total and phytate P release were evaluated using two-way ANOVA, with digestion stage (acid, alkaline, or total) and diets as factors. In vitro assay data of pentoses and amino acids release for each digestion stage (acid, alkaline, or total) were analyzed by two-way ANOVA, with diets and fish digestive enzymes as factors, followed by Bonferroni’s multiple range test to detect significant differences among means (*p* < 0.05).

Data from in vivo digestibility assay was analyzed by one-way ANOVA, followed by Bonferroni’s multiple range test to detect significant differences among means (*p* < 0.05). All statistical analyses were conducted using *IBM SPSS Statistics* software version 26 (IBM, NY, USA; https://www.ibm.com/products/spss-statistics).

## Results

### Enzyme production by SSF of BSG

SSF of BSG with *Aspergillus ibericus* allowed the production of 50 g of lyophilized SSF-BSG extract per kg of BSG. The lyophilized SSF-BSG extract had 15,885 and 1343 U g^−1^ of xylanase and cellulase activities, respectively. The enzyme activity of the experimental diets reflected the amount of lyophilized SSF-BSG extract added to the diets. The NAT supplemented diet had no protease activity, and cellulase and xylanase activities of this diet were within the range values of the BSG0.2 diet, while β-glucosidase and phytase activities were within the range values of the BSG0.4 diet (Table 1).

### In vitro assays

**Figure 2:**
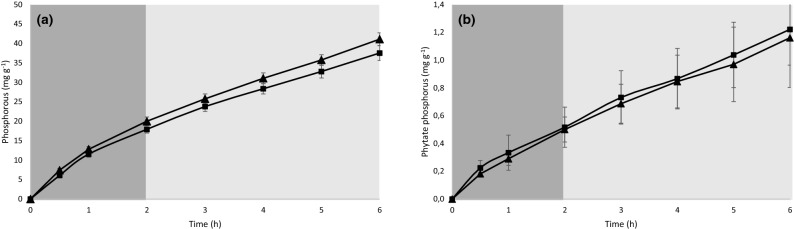
Total phosphorus (**a**) and phytate phosphorus (**b**) release (mg g^−1^ of diet) from control (filled black square) and BSG0.4 (filled black triangle) diets under the simulated conditions of European seabass digestive tract. Stomach and intestinal digestion are represented in dark and light grey, respectively. Values are presented as the mean of the cumulative values ± standard deviation.

In Assay 1, aimed to evaluate differences in bioavailability of total and phytate P for control and BSG0.4 diets, a steady release of total and phytate P was observed with time for both diets. However, the inclusion of 0.4% of SSF-BSG extract in the diet significantly increased the release of total P (Fig. 2, Table 3).

**Table 3:** Total and phytate phosphorus release of control and BSG0.4 diets during in vitro digestion (assay 1).

Diet	Digestion stage	Phosphorus (mg g^−1^ diet^−1^)		Phytate phosphorus (mg g^−1^ diet^−1^)		
		Mean ± SD		Mean ± SD		
Control	Acid	17.9 ± 0.68		0.52 ± 0.10		
	Alkaline	19.6 ± 0.72		0.36 ± 0.03		
	Total digestion	37.5 ± 1.40		1.22 ± 0.18		
BSG0.4	Acid	20.0 ± 0.77		0.50 ± 0.06		
	Alkaline	21.1 ± 0.40		0.32 ± 0.13		
	Total digestion	41.1 ± 1.20		1.16 ± 0.25		

Two-way ANOVA*	Phosphorus			Phytate phosphorus		
	Diet	Digestion stage	Interaction	Diet	Digestion stage	Interaction
	*	n.s.	n.s.	n.s.	n.s.	n.s.

Values presented as mean (n = 3) and standard deviation (SD).

Two-way ANOVA testing for the effect of the diet (control and BSG0.4) and digestion stage (acid, alkaline and total).

*p < 0.05; **p < 0.01; ***p < 0.001; n.s.: not significant.

**Figure 3:**
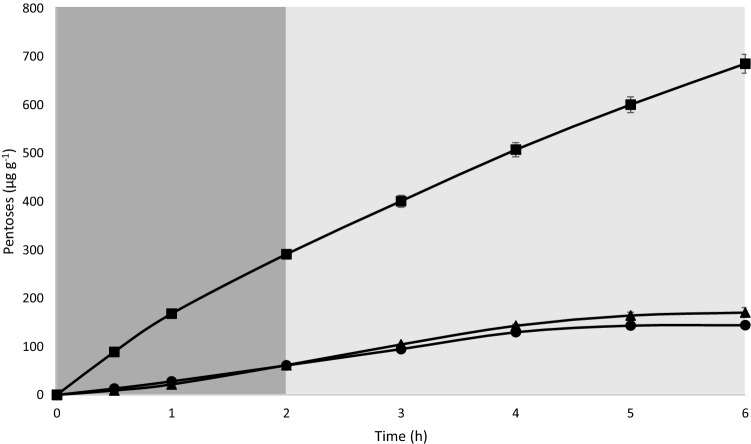
Pentoses release (µg g^−1^ of diet) from BSG0.4 diet with inactivate fish enzymes (filled black square), inactive BSG 0.4 diet with active fish enzymes (filled black triangle) and both inactive BSG 0.4 diet and fish enzymes (filled black cirle), under the simulated conditions of European seabass digestive tract. Stomach and intestinal digestion are represented in dark and light grey, respectively. Values are presented as the mean of the cumulative values ± standard deviation.

In Assay 2, designed to assess the interaction effect between endogenous (fish) and exogenous (SSF-BSG extract) enzymes on BSG0.4 diet carbohydrates hydrolysis, it was observed (i) a basal release of pentoses from the diet in the absence of any active enzymes; (ii) no significant effect of fish enzymes on such release; (iii) a fourfold increase in pentoses release with active relative to inactive BSG0.4 diet and; (iv) a 14% reduction on pentoses release when BSG0.4 diet was mixed with active fish enzymes compared to the amount released when fish enzymes were inactivated (Table 4). The dynamic of pentoses release during the in vitro hydrolysis is shown in Fig. 3.

**Table 4:** Pentoses release (µg g^−1^ diet h^−1^) of BSG0.4 diet during the gastric, intestinal, and total in vitro digestion.

Digestion stage	Inactive diet BSG0.4 *plus* inactive fish enzymes	Inactive diet BSG0.4 *plus* active fish enzymes	Active diet BSG0.4 *plus* inactive fish enzymes	Active diet BSG0.4 *plus* active fish enzymes
Acid	61.6 ± 0.55^b^	61.0 ± 1.64^b^	291.2 ± 6.50^a^	262.0 ± 27.5^a^
Alkaline	108.8 ± 9.54^c^	83.2 ± 1.39^c^	394.3 ± 7.52^a^	328.3 ± 15.1^b^
Total digestion	170.4 ± 10.1^b^	144.2 ± 3.03^b^	685.5 ± 13.84^a^	590.3 ± 42.0^a^

Values presented as mean (n = 3) ± standard deviation (SD).

Values in the same row with different subscripts are significantly different (*p* < 0.05).

**Figure 4:**
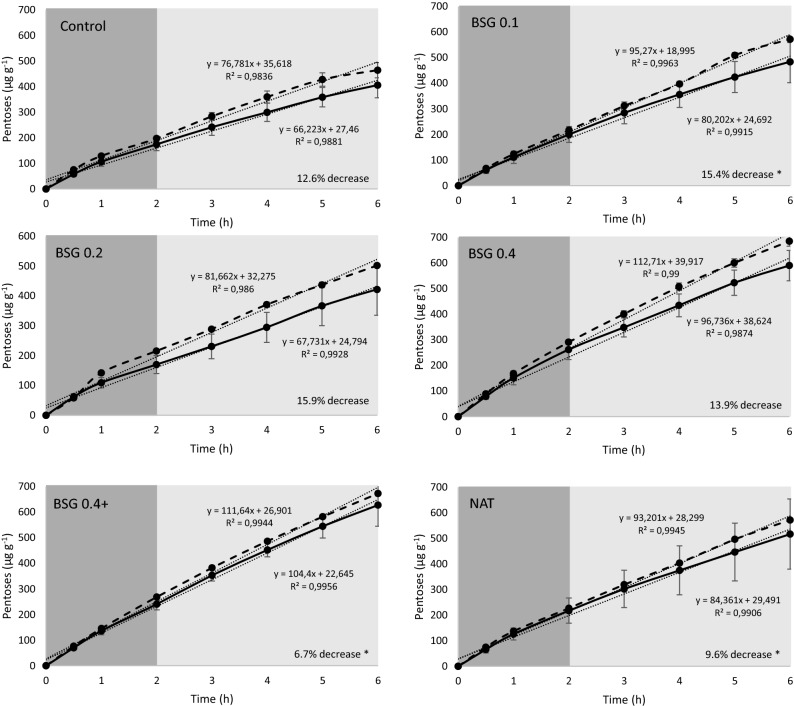
Pentoses release (mg g^−1^ of diet) from the different experimental diets under the simulated conditions of the European seabass digestive tract with active (continuous line) and inactive fish enzymes (dashed line). The slope of linear fitting provides the rate of release of pentoses per hour (mg g^−1^ of diet h^−1^). Stomach and intestinal digestions are represented in dark and light grey, respectively. Values expressed as mean ± standard deviation. Asterisks indicate significant differences between active and inactive fish enzymes (*p* < 0.05).

**Figure 5:**
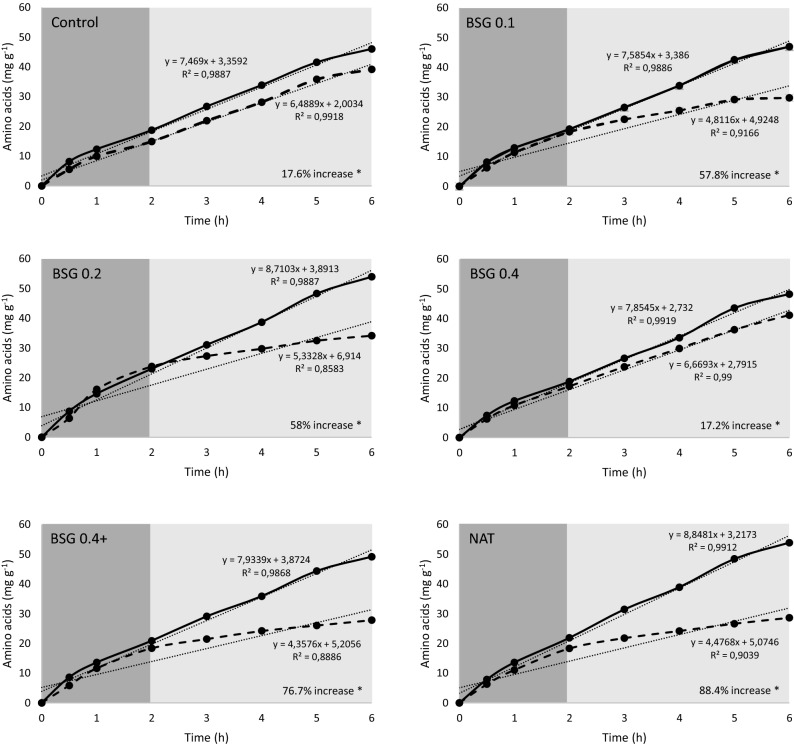
Amino acids release (mg g^−1^ of diet) from the different experimental diets under the simulated conditions of the European seabass digestive tract with active (continuous line) and inactive fish enzymes (dashed line). The slope of linear fitting provides the rate of release of amino acids per hour (mg g^−1^ of diet h^−1^). Stomach and intestinal digestions are represented in dark and light grey, respectively. Values expressed as mean ± standard deviation. Asterisks indicate significant differences between active and inactive fish enzymes (p < 0.05).

In Assay 3, designed to evaluate the effect of dietary inclusion of SSF-BSG extract or Natugrain on the hydrolysis of carbohydrates and protein in all experimental diets, it was observed (i) a positive effect of the supplementation with SSF-BSG extract on the release of pentoses from the control diet; (ii) a linear increase of pentoses release with the increase of dietary SSF-BSG extract supplementation level (R = 0.75; P < 0.05); (iii) no additional improvement on carbohydrates or protein hydrolysis when the diet containing 0.4% of SSF-BSG extract was combined with Silica+ and a plant-based detoxifier Ena-Detox; (iv) pentoses or amino acids release of Natugrain supplemented diet was similar to that measured in the control and SSF-BSG extract supplemented diets; (v) an interactive effect of active fish enzymes and SSF-BSG extract, decreasing carbohydrates hydrolysis by 7% (BSG0.4+ diet) and 16% (BSG0.2 diet); vi) an interactive effect of active fish enzymes and diets, increasing protein hydrolysis by 18% (control and BSG0.4% diets), 58% (BSG0.1 and BSG0.2 diets), 77% (BSG0.4+ diet), and 88% (NAT diets) (Tables 5 and 6; Figs. 4 and 5).

**Table 5:** Pentoses release (µg g^−1^ diet h^−1^) during the gastric, intestinal, and total in vitro digestion.

Diet		Fish enzymes		Acid digestion		Alkaline digestion		Total digestion	
Control		Active		174.3 ± 17.5		231.5 ± 18.0		405.8 ± 35.2	
		Inactive		197.2 ± 6.3		267.0 ± 15.9		464.2 ± 21.2	
BSG0.1		Active		200.1 ± 22.0		284.1 ± 36.8		484.1 ± 58.1	
		Inactive		217.1 ± 4.3		355.2 ± 5.8		572.3 ± 9.2	
BSG0.2		Active		170.0 ± 21.5		251.8 ± 41.7		421.8 ± 61.4	
		Inactive		215.5 ± 0.8		286.2 ± 3.6		501.6 ± 3.8	
BSG0.4		Active		262.0 ± 27.5		328.3 ± 15.1		590.3 ± 42.0	
		Inactive		291.2 ± 6.5		394.3 ± 7.6		685.5 ± 13.8	
BSG0.4+		Active		240.8 ± 16.0		386.8 ± 42.6		627.6 ± 58.6	
		Inactive		268.7 ± 3.0		404.3 ± 3.8		673.0 ± 6.5	
NAT		Active		217.4 ± 34.8		299.3 ± 62.4		516.7 ± 97.0	
		Inactive		227.1 ± 1.4		344.4 ± 3.8		571.5 ± 3.8	

Two-way ANOVA	Variance source			Diets					
	Diet	Fish enzymes	Interaction	Control	BSG0.1	BSG0.2	BSG0.4	BSG0.4+	NAT
Acid	***	*	n.s.	c	bc	c	a	ab	abc
Alkaline	***	*	n.s.	c	abc	bc	ab	a	abc
Total	***	*	n.s.	b	ab	b	a	a	ab

Values presented as mean (n = 3) and standard deviation (SD).

Two-way ANOVA testing for the effect of the diet (control, BSG0.1, BSG0.2, BSG0.4, BSG0.4 + ; NAT) and fish enzymes (active and inactive): *p < 0.05; ***p < 0.001; n.s.: not significant.

**Table 6:** Amino acids release (mg g^−1^ diet h^−1^) during the gastric, intestinal, and total in vitro digestion.

Diet		Fish enzymes		Acid digestion		Alkaline digestion		Total digestion	
Control		Active		18.7 ± 0.06		27.3 ± 0.29 ^c^		46.1 ± 0.36^c^	
		Inactive		14.9 ± 0.09		24.3 ± 0.13 ^A^		39.2 ± 0.05^AB^	
BSG0.1		Active		19.2 ± 0.16		27.7 ± 0.31 ^c^		47.0 ± 0.15^bc^	
		Inactive		18.3 ± 0.21		11.4 ± 0.13^B^		29.7 ± 0.21^BC^	
BSG0.2		Active		23.0 ± 0.02		31.0 ± 0.22^ab^		54.0 ± 0.20^a^	
		Inactive		23.8 ± 3.5		10.4 ± 0.45^B^		34.2 ± 3.00^ABC^	
BSG0.4		Active		18.9 ± 0.01		29.4 ± 0.07^bc^		48.3 ± 0.06^bc^	
		Inactive		17.3 ± 0.17		23.9 ± 0.37^A^		41.2 ± 0.20^A^	
BSG0.4+		Active		20.9 ± 0.3		28.3 ± 0.21^c^		49.2 ± 0.53^b^	
		Inactive		18.4 ± 0.51		9.4 ± 0.06^B^		27.8 ± 0.58^C^	
NAT		Active		21.9 ± 0.04		32.0 ± 0.36^a^		53.9 ± 0.32^a^	
		Inactive		18.3 ± 0.12		10.3 ± 0.26^B^		28.6 ± 0.13^C^	

Two-way ANOVA	Variance source			Diets					
	Diet	Fish enzymes	Interaction	Control	BSG0.1	BSG0.2	BSG0.4	BSG0.4+	NAT
Acid	***	*	n.s.	b	ab	b	ab	ab	ab
Alkaline	***	*	**						
Total	**	***	***						

Values presented as mean (n = 3) and standard deviation (SD).

Two-way ANOVA testing for the effect of the diets (control, BSG0.1, BSG0.2, BSG0.4, BSG0.4 + ; NAT) and fish enzymes (active and inactive): *p < 0.05; **p < 0.01; ***p < 0.001; n.s.: not significant. If the interaction was significant, one-way ANOVA was performed testing the effect of the diets during digestion with active (lower letters) or inactive fish enzymes (upper letters).

### In vivo digestibility assay

The digestibility of dry matter, starch, cellulose, and glucans was significantly higher with the BSG0.4 diet than with the BSG0.1 or control diets. Energy digestibility was higher with the BSG0.4 diet than the control diet, but it was not significantly different from the BSG0.1 diet. The digestibility of hemicellulose, xylan, and arabinan tended to be higher with the BSG0.4 diet than in the other dietary treatments, though differences were not statistically significant (Table 7)*.*

**Table 7:** Apparent digestibility coefficients (ADC) of dietary nutrients and energy in European seabass juveniles fed with the experimental diets.

Diets	Control	BSG 0.1	BSG 0.4
Dry matter	67.7 ± 0.04^b^	68.0 ± 1.61^b^	72.8 ± 2.08^a^
Protein	96.2 ± 0.26	96.4 ± 0.43	96.4 ± 0.46
Starch	75.8 ± 3.17^b^	80.1 ± 0.31^b^	82.4 ± 1.88^a^
Energy	78.3 ± 1.69^b^	79.9 ± 1.28^ab^	83.4 ± 1.12^a^
Hemicellulose	49.9 ± 2.09	49.7 ± 6.70	58.0 ± 2.04
Cellulose	16.4 ± 1.14^b^	14.7 ± 2.85^b^	25.5 ± 4.79^a^
Lignin	0.0	0.0	0.0
Glucan	16.4 ± 1.14^b^	14.7 ± 2.85^b^	25.5 ± 4.79^a^
Xylan	46.1 ± 3.49	45.4 ± 6.50	56.0 ± 4.50
Arabinan	56.3 ± 0.20	57.1 ± 8.71	63.1 ± 1.03

Values presented as mean (n = 3) and standard deviation (SD).

Values in the same row with different subscripts are significantly different (*p* < 0.05).

## Discussion

### Enzyme production by SSF of BSG

BSG is an underutilized agro-industrial by-product with the potential to replace high carbon footprint conventional feedstocks, given its high production rate, acquisition price, and supply chain^42^. However, for the moment, no “established market function” for BSG exist, and its high moisture content and low sugars content hinder the development of economic models for its valorisation^43^. Considering costs associated with BSG disposal, new and promising utilizations for this by-product must be envisioned to meet circular economy guidelines and ensure sustainability and food security. New and alternative applications for BSG are increasing, as construction materials, alternative fuels, textile applications, human food, and animal feeds^44^.

The reutilization of BSG towards the obtention of value-added compounds following eco-friendly and circular economy approaches, such as SSF, is of utmost importance. BSG is a lignocellulosic by-product with 24% of hemicellulose and 21% of cellulose, with a high potential to produce carbohydrases by SSF, an under-explored process. In the present study, the biotechnological treatment of BSG through SSF with *A. ibericus* resulted in high xylanase and cellulase production (15,885 and 1343 U g^−1^, respectively). Previously, SSF of BSG with *Penicillium janczewskii* produced 371 U xylanase g^−1^ BSG^23^, a lower value than obtained in the present study. These results confirm the potential of agro-industrial by-products to produce added-value compounds, such as enzymes, through SSF processes^45–47^.

The production of enzymes through SSF processes presents several advantages compared to SmF, including higher enzymatic productivity and stability within a broader range of conditions, and lower susceptibility to inactivation by the substrate^15^. The present study confirmed that the enzymes of SSF-BSG extract remained functional throughout the acid and alkaline digestion. Indeed, even though endogenous fish enzymes may negatively interact with the SSF-BSG extract activity, the in vitro carbohydrates bioavailability increased fourfold when comparing an active versus inactive SSF-BSG extract supplemented diet (BSG0.4 diet). Accordingly, Vasconcellos et al*.* compared the production of endoglucanase and β-glucosidase through SSF or SmF and concluded that SSF produced enzymes less susceptible to inactivation by the substrate's phenolic compounds and that were highly thermostable, maintaining 80% to 100% of activity after incubation at 50 °C for 24 h^48^.

### In vitro and in vivo assays

Experimental approaches based on in vitro models simulating biological functions are increasingly encouraged to reduce the number of animals used for experimental purposes^27,49^ and are in line with the eco-friendly approach of the present study. In vitro assays simulating the European seabass gut biochemical conditions and previously optimized for this species^27,28^, allowed a detailed assessment of the effect of SSF-BSG extract and Natugrain on nutrients bioavailability, the interactive effects of SSF-BSG extract and Natugrain with fish enzymes during digestion, and the fine-tune selection of the most potential experimental diets to be further evaluated during an in vivo digestibility trial.

Phytate P is present in a wide range of plant feedstuffs but is unavailable for most fish species as they lack intestinal phytases, impairing mineral utilization, growth, and feed utilization. Phytate P can bind with minerals, such as calcium, zinc, sodium, and others, decreasing its solubility and, concomitantly, minerals bioavailability^50^. Dietary supplementation with phytase has been used as a nutritional strategy to increase phosphorus bioavailability for fish and other animals^50^. Even though *A. ibericus* used in the present study was selected based on its high capacity to produce carbohydrases, phytases were also produced during SSF of BSG. In vitro assay 1 confirms that supplementation of a plant-based diet with 0.4% of SSF-BSG extract significantly increased total P release but not that of phytate P. This result may be interpreted as an increase in the potential bioavailability of this element.

In vitro digestibility assay 2 confirms the potential of SSF-BSG extract to hydrolyze dietary carbohydrates. Dietary inclusion of 0.4% SSF-BSG extract (BSG0.4 diet) increased more than fourfold the pentoses release during in vitro digestion compared to the same diet in which the enzymatic activity was previously inactivated by heat. Moreover, combining active or inactive BSG0.4 diet with inactive fish enzymes during in vitro digestion did not affect pentoses release, corroborating the main role of SSF-BSG extract on carbohydrates hydrolysis. However, combining active BSG0.4 diet with active fish enzymes during in vitro acid or alkaline digestion decreased pentoses release by about 10% to 16%, respectively, suggesting a negative interaction between endogenous fish enzymes and SSF-BSG extract carbohydrases. The efficacy of feed enzymes is affected by different factors, including pH specificity and gastrointestinal inhibitors, limiting the potential benefits of dietary supplementation with exogenous enzymes^4,50^. For example, Morales et al*.* observed that both activity and stability of a fungal phytase (from *Peniophora lycii*) were significantly compromised when evaluating the in vitro acid digestion of rainbow trout (*Oncorhynchus mykiss*)^51^.

Moreover, in assay 2, the in vitro digestion of inactive BSG0.4 diet combined with inactive endogenous fish enzymes also resulted in pentoses release. Pentoses release without the presence of active carbohydrases indicates that some non-starch glucosidases may be present in plant feedstuffs and retained some activity after pelleting and digestion processes. In fact, cellulases have been found in various plants, being involved in the defense mechanisms, lignification process, cell-wall development, and symbiotic activities of the plant^52,53^.

The in vitro digestibility assay 3 confirms a dose–response effect of SSF-BSG extract on carbohydrates hydrolysis, as pentoses release linearly increased with dietary SSF-BSG extract inclusion level (R = 0.75; P < 0.05, including total digestion data with active or inactive fish enzymes). Moreover, dietary supplementation with 0.4% SSF-BSG extract combined with the commercial catalyzer, Silica+, and a plant-based detoxifier, Ena-Detox, promoted similar carbohydrates or protein hydrolysis than to the diet supplemented with 0.4% SSF-BSG extract alone. Contrarily to the present results, silicon dioxide has been utilized in poultry and pigs diets with promising results on growth performance and feed utilization^54,55^.

Additionally, the in vitro digestibility assay 3 confirmed that SSF-BSG extract enzymes exhibited carbohydrates and protein hydrolytic activity at both acid and alkaline pH (5 and 8.5, respectively), being more efficient during the alkaline than the acid stage. Although optimum enzyme activity occurs under certain pH and temperature conditions^8^, the higher carbohydrates hydrolysis observed during the alkaline digestion is not aligned to the optimum pH for maximum activity of carbohydrases produced in SSF by filamentous fungi. In general, cellulases produced by filamentous fungi have maximum activities at acidic pH (3.6–5)^56^. For example, xylanases produced by *A. fumigatus* showed higher activity at pH between 3 and 6^57^, and xylanases and β-glucosidases produced through the SSF of *Nopalea cochenillifera* with *A. niger* and *Rhizopus* sp. presented optimum activities at pH between 4 and 7^58^. Besides the pH and temperature effect, enzymes activity may also be affected by enzymes accessibility to the substrate. In the present study, the higher pentoses and amino acids release observed in the alkaline stage may have been triggered by the previous stomach digestion, which facilitated protein and carbohydrate hydrolysis during intestinal digestion by increasing substrate accessibility to the enzymes. Similarly, other authors also observed that the acid pre-digestion of fishmeal and plant feedstuffs increased intestinal protein hydrolysis^27,59^, reflecting the significant contribution of alkaline digestion in the total protein hydrolysis^60^.

In vitro trials are well suited to evaluate differences in the bioaccessibility of nutrients and the effect of diverse factors that may influence their potential bioavailability upon intestinal absorption. For this reason, in vitro trials provide a pre-absorption estimation of nutrient bioavailability, while in vivo digestibility measures are considered a post-absorption estimation of nutrients bioavailability, being affected by other factors such as microbial biomass or products of their metabolism present in fecal mass^28^. This could explain why not always both types of determinations present a good correlation. Nevertheless, the results of the in vivo digestibility trial of the present study corroborated to a great extent the results obtained in vitro. In both trials, dietary SSF-BSG extract supplementation did not significantly affect protein hydrolysis, whereas bioavailability of carbohydrates increased with 0.4% SSF-BSG extract incorporation. Dietary supplementation with 0.4% of SSF-BSG extract increased the pentoses release by circa 45% in the in vitro digestibility trial. Accordingly, in the in vivo digestibility trial, 0.4% SSF-BSG extract supplementation increased cellulose and hemicellulose digestibility by circa 52% and 16%, respectively, while xylan, glucan, and arabinan digestibility increased 20%, 16%, and 11%, respectively. For carnivorous fish, as European seabass, the digestibility of NSPs was never evaluated. Nevertheless, the digestion of some NSPs fractions has been reported for some non-carnivorous fish species. For example, in Nile tilapia (*Oreochromis niloticu*s), depending on dietary fiber composition, NSPs digestibility averaged 24%^61^, ranging between 23 and 73%^62,63^, with soluble NSPs digestibility being 60% and insoluble NSPs undigested^64^. The beneficial effect of dietary supplementation with SSF-BSG extract on carbohydrates bioavailability of European seabass is probably not only related to NSPs digestion. The effect of SSF-BSG extract carbohydrases on the digesta physical properties, decreasing its viscosity, releasing entrapped nutrients by facilitating the fish enzymes action^4,7^, and promoting microbiota fermentation processes^3,61,65^ may also have contributed to the present results. Fish microbiota plays a significant role in digestion and metabolism^66^ and is modulated by dietary supplementation with exoenzymes, supporting the development of a more favorable microbiota community for nutrient hydrolysis^6,67^.

Present results confirmed that dietary incorporation of SSF-BSG extract is a promising nutritional tool to increase nutrient digestibility by hydrolyzing NSPs. The use of carbohydrases in fish feeds was revised by Castillo and Gatlin and Zheng et al*.* with promising results^4,8^. For example, in European seabass, dietary supplementation with a commercial carbohydrases complex increased dry matter, protein, lipids, energy, and phosphorus digestibilities, decreased chyme pH along the intestine but did not affect digestive enzymes activity^68^. In white seabream (*Diplodus sargus*), the same carbohydrases complex increased feed, nitrogen, and energy utilization efficiency as well as intestinal amylase and lipase activities^5^. This carbohydrases complex also increased dry matter and some amino acids digestibility, intestinal lipase, and protease activity, and modulated intestinal microbiota in turbot (*Scophthalmus maximus*)^6^. Dietary supplementation with cellulase in a *Chlorella*-based diet increased dry matter, energy, and protein digestibility, digestive enzymes activity, and growth performance of crucian carp (*Carassius auratus*)^69^. In juvenile yellow croaker (*Larimichthys crocea*), dietary supplementation with xylanase improved zootechnical performance, intestinal morphology structure, and microbiota constitution^70^. An enzymatic-rich extract produced from SSF of agro-industrial by-products with *Aspergillus niger* and *A. oryzae* increased protein, mineral, energy, and lipids digestibility of Nile tilapia^71^.

## Conclusions

SSF of BSG with *Aspergillus ibericus* allowed the production of highly active enzymatic enriched extract. The in vitro assays confirmed that the inclusion of 0.4% SSF-BSG extract in a plant-based diet for European seabass increases phosphorus and carbohydrates hydrolysis, being more effective during the alkaline than the acid stage of digestion. Fish endogenous enzymes reduce the efficacy of SSF-BSG extract by about 7% to 16%. The in vivo digestibility assay results corroborated the data obtained in in vitro assays. Dietary supplementation with 0.4% SSF-BSG extract increased digestibility of dry matter, energy, starch, cellulose, and glucans. This study also highlighted the partial digestibility of NSPs in plant-based diets for carnivorous fish, requiring further research.

Overall, the results showed the potential of SSF of BSG to produce an added-value enzymatic supplement that increases the bioavailability of plant ingredients. Additionally, the use of BSG, an underutilized agro-industrial by-product, as substrate for filamentous fungi enzymes production, represents a synergy between economic and environmental efforts under the circular economy guidelines. Further research is needed to develop technological strategies to incorporate the SSF-BSG extract into aquafeeds, including coating, to reduce the negative interaction with fish enzymes, and assess the economic and environmental impact of SSF-BSG extract utilization.

## Data Availability

The datasets obtained and analyzed in the present study are not available for public consultation.

## Abbreviations

BSG: Brewer’s spent grain
SSF: Solid-state fermentation
NAT: Natugrain
NSPs: Non-starch polysaccharides
ADC: Apparent digestibility coefficient
